# Impaired Abcb1a function and red meat in a translational colitis mouse model induces inflammation and alters microbiota composition

**DOI:** 10.3389/fmed.2023.1200317

**Published:** 2023-07-31

**Authors:** Allan Stensballe, Tue Bjerg Bennike, Gitte Ravn-Haren, Alicja Mortensen, Christopher Aboo, Lina Almind Knudsen, Malte C. Rühlemann, Svend Birkelund, Corinne Bang, Andre Franke, Ulla Vogel, Axel Kornerup Hansen, Vibeke Andersen

**Affiliations:** ^1^Department of Health Science and Technology, Aalborg University, Aalborg, Denmark; ^2^Clinical Cancer Research Center, Aalborg University Hospital, Aalborg, Denmark; ^3^National Food Institute, Technical University of Denmark, Lyngby, Denmark; ^4^National Research Centre for the Working Environment, Copenhagen, Denmark; ^5^Sino-Danish Center for Research and Education, University of Chinese Academy of Sciences, Beijing, China; ^6^Institute of Regional Health Research-Center Soenderjylland, University of Southern Denmark, Odense, Denmark; ^7^Institute of Clinical Molecular Biology, Christian-Albrechts-Universität zu Kiel, Kiel, Germany; ^8^Department of Veterinary and Animal Sciences, University of Copenhagen, Copenhagen, Denmark; ^9^Molecular Diagnostic and Clinical Research Unit, University Hospital of Southern Denmark, Aabenraa, Denmark; ^10^Institute of Molecular Medicine, University of Southern Denmark, Odense, Denmark

**Keywords:** Abcb1, colon mucosa, proteomics, p-glycoprotein, diet, microbiota, NETs, neutrophil degranulation

## Abstract

Inflammatory Bowel Disease (IBD) affects approximately 0.3% of the global population, with incidence rates rising dramatically worldwide. Emerging evidence points to an interplay between exposome factors such as diet and gut microbiota, host genetics, and the immune system as crucial elements in IBD development. ATP-binding cassette (ABC) transporters, including human p-glycoprotein encoded by the Abcb1 gene, influence intestinal inflammation, and their expression may interact with environmental factors such as diet and gut microbes. Our study aimed to examine the impact of protein sources on a genetic colitis mouse model.

**Methods:**

Abcb1a-deficient colitis mice were fed either casein or red meat-supplemented diets to investigate potential colitis-aggravating components in red meat and their effects on host-microbiota interactions. We conducted deep label free quantitative proteomic inflammation profiling of gastrointestinal tissue (colon, ileum) and urine, and determined the overall microbiome in feces using 16S rRNA gene sequencing. Microbiota shifts by diet and protein transporter impairment were addressed by multivariate statistical analysis. Colon and systemic gut inflammation were validated through histology and immune assays, respectively.

**Results:**

A quantitative discovery based proteomic analysis of intestinal tissue and urine revealed associations between ileum and urine proteomes in relation to Abcb1a deficiency. The absence of Abcb1a efflux pump function and diet-induced intestinal inflammation impacted multiple systemic immune processes, including extensive neutrophil extracellular trap (NET) components observed in relation to neutrophil degranulation throughout the gastrointestinal tract. The colitis model’s microbiome differed significantly from that of wild-type mice, indicating the substantial influence of efflux transporter deficiency on microbiota.

**Conclusion:**

The proteomic and microbiota analyzes of a well-established murine model enabled the correlation of gastrointestinal interactions not readily identifiable in human cohorts. Insights into dysregulated biological pathways in this disease model might offer translational biomarkers based on NETs and improved understanding of IBD pathogenesis in human patients. Our findings demonstrate that drug transporter deficiency induces substantial changes in the microbiota, leading to increased levels of IBD-associated strains and resulting in intestinal inflammation.

**GRAPHICAL ABSTRACT:**
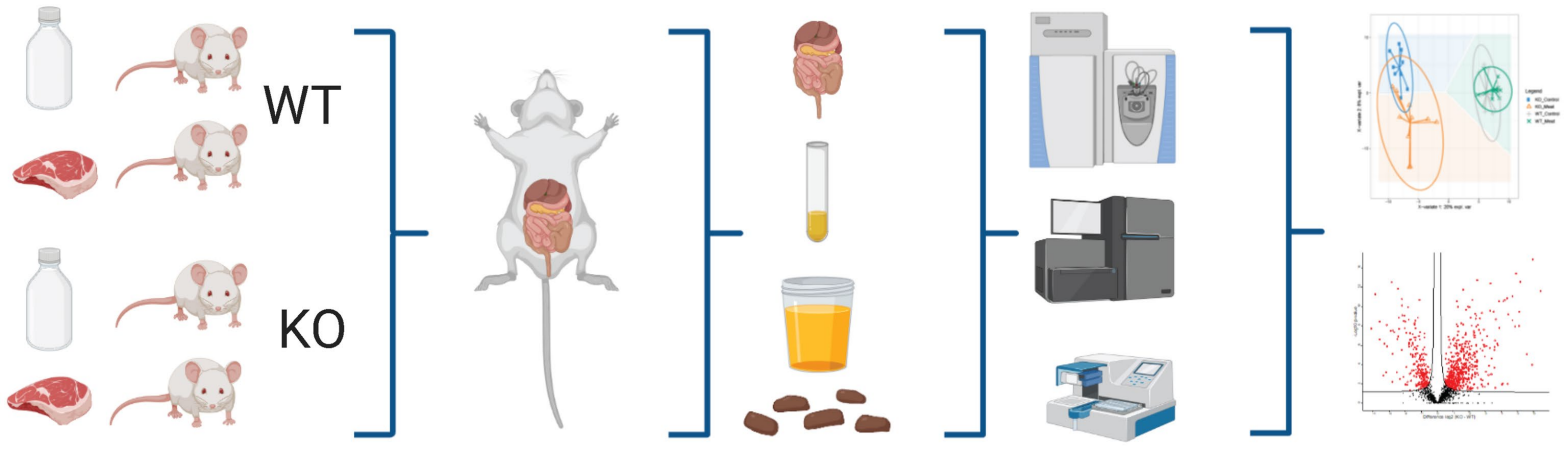

## Introduction

Inflammatory Bowel Disease (IBD), including ulcerative colitis (1) (UC) and Crohn’s disease (CD) (2), affects more than 0.3% of the world’s population, or > 20 million people, and the incidence is increasing dramatically globally (3). The disease typically develops early in life and markedly affects the life quality of patients and their families, and represents a significant burden on society due to, among others, lost educational opportunities, absenteeism, and health care expenses. Thus, there is a high demand for improved prevention and management of this complex and costly disease.

Conceptual nutritional trials now corroborate the inflammatory nature of a Western diet in IBD, supporting the concept of diet-induced metabolic gut inflammation (4). Emerging evidence suggests an interaction between the environment (exposome), such as diet, gut microbiota or host genetics, and the immune system plays an essential role in the development of IBD (5–7).

We have previously extensively reviewed the clinical impact of red and processed meat intake (8). Prospective cohort studies conclude that high consumption of red and processed meat is associated with an increased risk of IBD, particularly UC and colorectal cancer (9, 10). The original investigations of this translational mouse model indicate that the selected model may provide new insight into the pathogenesis of IBD and colorectal cancer as well as the nature of dysregulated immune reactivity to intestinal microbiota (11).

Genetic variations of the innate and adaptive immune systems are associated with IBD susceptibility but explain only a minor part of disease susceptibility (12). Given the multifactorial etiology of IBD and the continued lack of insight into dietary protein source’s effect on disease etiology and microbiota, genetic variation needs to be integrated into one model to capture reliable biological markers with diagnostic and prognostic value. In IBD, a lower fecal microbiota diversity has consistently been found compared with healthy subjects (13) and relates to disease phenotypes (14). In addition, it has been suggested that a Western-style diet, such as those high in meat intake, confers a higher risk for IBD, although evidence for this is limited (15). An imbalance of microbial homeostasis may lead to the colonization and invasion of opportunistic pathogens in the gut, which increases the risk of the host immune response and promotes the development of IBD (16, 17). Neutrophil extracellular traps (NETs) (18, 19) containing extracellular enzymes, antimicrobial peptides and DNA are now recognized as an important host response in patients with UC (20) and were suggested as a potential new biomarker for tracking disease activity and associated with microbiota interactions. Multiple NETs components are associated with reactions to autoantibodies (21). Animal models have proven effective when investigating complex biological mechanisms potentially transferable to humans (22–27).

ATP-binding cassette (ABC) transporters are a family of integral membrane proteins that translocate substrates across cellular membranes using ATP (28). ABC transporters, including p-glycoprotein (MDR1) may affect intestinal inflammation and potentially interact with the environment, such as diet and gut microbiota compositions. Among CD and UC patients the Abcb1/MDR1 gene have been determined as risk factor to the latter (29). Among ABC transporters, the murine genetic Abcb1a (Mdr1a) knockout (KO) mouse may serve as a colitis model as a proportion of the mice develop colitis when exposed to commensal gut bacteria but not when the mice are maintained germ-free (11, 23). Therefore, this murine model provides a highly valuable IBD model for the study of essential initiating factors in IBD (24).

To identify potential diet-gut microbiome-host genetics and/or immune systems interactions relevant to translational investigations of patients with IBD, we used the murine Abcb1a deficient mouse colitis model. In addition, we fed the mice either casein or red meat supplemented diets to investigate potential colitis aggravating components in meat and the impact on host-microbiota. We hypothesize that this model can reveal microbiota-associated changes in the intestinal barrier related to the protein source. Dietary and genetic information, proteomics data and gut microbiota composition were integrated using multivariate statistical methods to highlight interactions between the different data layers.

## Materials and methods

### Animals and housing

A total of 31 FVB.129P2-Abcb1a^tm1Bor^N7 female KO mice and 30 FVB/NTac wild type (WT) female mice purchased as SPF animals from Taconic Europe A/S (Ll. Skensved, Denmark) were included in the present study (30). Animals (6 weeks of age at arrival) were ear marked and housed 4–5 per cage (based on body weight) in the same room. The mice were kept in type III cages (Scanbur A/S, Karlslunde) with Tapvei 4VH bedding, wooden blocks and hiding place (Brogaarden ApS, Lynge, Denmark) under controlled conditions (temperature 22 ± 1°C, relative humidity 55 ± 5%, 12/12 h light/dark cycle, air changes 8–10 times/h). Each group of animals (cages) were subject to identical environmental conditions including diet but excluding physical contact hence not direct microbiota transfer by, e.g., consumption of faces. The study was approved by the Danish Animal Experiments Inspectorate (license number 2012-15-2,934-00089) and was supervised by the in-house Animal Welfare Body of the National Food Institute at the Technical University of Denmark.

### Diets and study design

Minced beef meat with 4–7% fat was purchased from a local supermarket. Total fat content in the meat was assumed to be 4% based on data obtained from the Danish food composition database.^1^ The meat was lyophilized and stored at −20°C until delivery to Brogaarden ApS (Lynge, Denmark) for incorporation into powdered Altromin C 1000 diet prior to pelleting (Altromin GmbH, Lage, Germany). In the red meat containing diet (MEAT) 12% of the normal 17% casein content in Altromin C 1000 was replaced by the lyophilized minced beef meat. Altromin C 1000 (without red meat but with 17% casein) was used as a control (CTL) diet. Animals were offered acidified water to prevent growth of microorganisms and CTL or MEAT diets *ad libitum* during the treatment period of 20 weeks. Amino acid composition of CTL diet and MEAT diet is presented in Supplementary Table S1. After a 2-week acclimatization period KO and WT mice were randomly assigned to the MEAT and CTL groups: (1) CTL diet WT mice (*n* = 16), (2) MEAT diet WT mice (*n* = 15), (3) CTL diet KO mice (*n* = 16) and (4) MEAT diet KO mice (*n* = 14). All mice were observed twice a day for any abnormalities in clinical appearance. The weights of feed and water intakes were calculated as supplied minus remaining weekly for each cage throughout the treatment period. After 5 weeks (2 per group), 10 weeks (6 per group) and 20 weeks (4–6 per group) mice anesthetized with 1:1:2 mixture of Hypnorm (Fentanyl/Fluanison, VetaPharma, Leeds, United Kingdom) and Dormicum (Midazolam 5 mg/mL; Roche, Hvidovre, Denmark) were sacrificed by cervical dislocation. During the study two WT CTL, two WT MEAT, and two KO CTL mice died spontaneously and one WT CTL and two KO CTL mice were sacrificed due to disturbed clinical appearance.

### Bio fluids, feces, and tissue collection

#### Blood sampling

Prior to sacrifice blood was sampled from the periorbital sinus of anaesthetized (Hypnorm/Dormicum) animals into dry tubes. After 30 min, serum was isolated by centrifugation at 2500 g for 10 min and immediately frozen in liquid nitrogen and stored at −80°C until cell-free DNA (cfDNA) analysis.

#### Necropsy

The animals sacrificed at the scheduled terminations were subjected to a detailed necropsy (i.e., an examination of the external surfaces and orifices, of the thoracic and visceral cavities and of the organs *in situ* and after removal from the carcass). The genotype and diet groups were blinded during the *postmortem* examination. Abnormalities were noted and the relevant tissue samples were preserved in 4% neutral buffered formaldehyde or snap frozen in liquid nitrogen.

The animals sacrificed *in extremis* and the mice, which died spontaneously with autolysis not too advanced were necropsied but no samples were taken to any of analyzes.

#### Organ weights, intestinal tissue samples, fecal- and urinary collection

At the scheduled sacrifices, the following organs from each animal were dissected free of adjacent fat and connective tissue, and the weight was recorded: cecum (with and without content), liver, kidneys, heart, spleen and mesenteric lymph nodes. Samples from colon and small intestine, urine (24 h collected from 48 mice) and fresh feces (collected before placing the mice in cages for urine sampling) were snap-frozen in liquid nitrogen followed by storage of all biomaterials at −80°C. Tissue samples (colon, small intestine) and feces for quantitative polymerase chain reaction (qPCR) and Next generation sequencing were immediately after collection transferred to individual cryotubes prefilled with 0.5 mL RNAlater (Life Technologies, Carlsbad, CA, United States), stored at room temperature for 24 h followed by storage −80°C.

#### Histologic examination

From all animals killed on all scheduled sacrifices, colon Swiss Rolls were prepared according to the method of Moolenbeek and Rutenberg (1981) (31) with modifications. In brief, the entire colon (from the end of caecum to anus) was cut up longitudinally, the internal surface was cleaned with a 0.9% NaCl (saline) and the colon was spread on a polystyrene board. After an additional rinsing with saline, the tissue was covered with 10% formaldehyde for 5–10 min. Thereafter, the excess formaldehyde was rinsed with saline. The colon, starting from the cranial end and with a mucosal side upward, was rolled over a wooden stick soaked in saline, while avoiding stretching of the tissue. The end of each roll was fastened with a pin. Each roll was then placed in a small bucket with 10% formaldehyde and left overnight. After fixing, the rolls were processed, embedded in paraffin, sectioned in 5 μm thick slices, and stained with hematoxylin and eosin (H&E staining) for histological examination. Colon was examined (the genotype and diet groups blinded to observer), any changes were noted, and the incidence was recorded. Severity of changes was scored as described by Obermeier et al. (32) with modifications: Appearance of colonic mucosa (M): 0: normal morphology (thick mucosal folds, abundant goblet cells, no villi, only crypts); 1: loss of goblet cells; 2: loss of goblet cells in large area; 3: loss of crypts; 4: loss of crypts in large area. Infiltration of inflammatory cells (I): 0: no infiltration; 1: infiltrate around crypt basis; infiltrate reaching muscularis mucosa; 3: extensive infiltration reaching muscularis mucosa; 4: infiltration of submucosa (32). The total histological score represented the sum of mean group scores for appearance of mucosa and infiltration of inflammatory cells (total score = M + I).

### Sample preparation for proteomics (colon, urine, ileum)

Colon and ileum samples were prepared for proteomic analysis using an optimized filter-aided sample preparation protein digestion protocol as previously described (33). Essentially, the intestinal samples were homogenized in 0.5 mL cold lysis buffer (5% sodium deoxycholate, 50 mM triethylammonium bicarbonate, pH 8.5) using bead-beating (Bullet blender Gold; Next Advance; NY, United States) for 2 min, speed 9 using Stainless Steel Beads mix 0.9–2.0 mm. The protein concentration of the lysates was estimated by bicinchoninic acid assay with bovine serum albumin as standard, measured using microplate reader (Tecan, Männedorf, Switzerland). From each tissue and urine sample 100 μg total solubilized protein was reduced with 10 mM tris(2-carboxyethyl)phosphine (Thermo Scientific, Waltham, MA, United States) and alkylated using 50 mM 2-iodoacetamide (Sigma-Aldrich, St. Louis, MO, United States) in lysis buffer using 10 kDa spin-filter (YM-10; Millipore, Billerica, MA, United States). The proteins were digested to peptides overnight at 37°C with two μg sequencing grade modified trypsin (Promega, Madison, WI, United States). The peptide material was eluted from the spin-filter and purified by phase inversions with ethyl acetate and formic acid. The final peptide eluent was dried down in a vacuum centrifuge overnight and stored at −80°C until time of analysis.

For urine the protein fraction was precipitated by chloroform-methanol precipitation (34). 150 μL of methanol was added to 50 μL of urine sample in an Eppendorf Low bind tube, and then 50 μL of chloroform was added and lastly mixed with 200 μL of Milli-Q water. The samples were vortexed and centrifuged (15 min at 14,000 g) leading to two liquid phases, with the proteins precipitate in the interphase. The upper phase contained water soluble metabolites and the lower phase contained organic soluble compounds. In order to pellet the proteins, the upper phase was removed and discarded, 200 μL methanol was added, and the samples were centrifuged at 14,000 g for 15 min. The supernatant containing organic soluble compounds was removed and the protein pellet was air dried. The protein pellets were resuspended in 50 μL SDC (0.5% SDC in 50 mM TEAB) and the samples were vortexed. Finally, the samples were heated at 95°C for 5 min. Protein concentration was measured on an Implen NanoPhotometer using protein absorption method (Protein A280). The samples were stored at −80°C overnight.

### Proteomics–UPLC-TandemMS analysis

The protein lysates (colon, ileum, urine) were redissolved in 2% acetonitrile, 0.1% formic acid (Thermofisher Ultra) and the concentration of the redissolved peptide lysate was determined by measuring absorbance at 280 nm using a Nanophotometer (Thermo Scientific, Waltham, MA, United States). A total of 5 μg peptide material was analyzed per UPLC-TandemMS analysis and all samples were analyzed in technical duplicates in a randomized order of animals. The tryptic peptides were separated by a nanoUPLC system (RSLC;Thermo Scientific, Waltham, MA, United States) coupled online to a Q Exactive Plus mass spectrometer (Thermo Scientific, Waltham, MA, United States) using a reverse phase C18 material trapping column setup with 50 cm Acclaim PepMap100 main column at 40°C, approximately 550-650 bar (Thermo Scientific, Waltham, MA, United States). The liquid phase consisted of 98% solvent A (0.1% formic acid) and 2% solvent B (0.1% formic acid in acetonitrile), and the flow rate was kept constant at 300 nL/min. The peptides were eluted from the column by changing the liquid phase to 12% solvent B on a 5 min ramp gradient and subsequently to 35% solvent B on a 30 min ramp gradient. For validation of Abcb1a expression, four KO and four WT samples were analyzed on an extended (120 min) gradient to increase sensitivity. The eluting peptides were introduced directly into the mass spectrometer by a picotip emitter for nanoflow ionization (New objective, Woburn, MA, United States). The mass spectrometer was operated in positive mode using a data-dependent acquisition method, selecting up to 12 MS/MS acquisitions of eluting ions in the m/z 350–1,400 range based on highest signal intensity for fragmentation.

### Proteomics–data preprocessing

The measured peptide signal intensities from the full MS scans were integrated using MaxQuant 1.6.0.2 software (35), and used to calculate the relative protein quantities using label-free quantification (LFQ). The fragment MS/MS scans were searched against the Uniprot *Mus musculus* C57BL/6 J Reference proteome (UP000000589). The following abundant peptide modifications were included in the analysis: carbamidomethylated cysteine residues (fixed), acetylation of N peptides from the N-terminal of proteins (variable) and oxidation of methionine (variable).

A target-decoy fragment spectra search strategy was employed in MaxQuant and used to filter the identified proteins and peptides to below 1% false discovery rate (FDR). An LFQ analysis was performed in MaxQuant of all proteins, part from Abcb1 which was quantified using MS2 counts. The list of proteins and LFQ-values were imported to Perseus 1.6.0.2. The raw MS data and MaxQuant search output have been deposited to the ProteomeXchange Consortium^2^ with the dataset identifier PXD016223 (36).

The replicates were averaged by the median. The list of identified proteins was further reduced by filtering to ensure high quality quantitative data: (1). The quantitation of any protein was required to be based on at least two quantifiable peptides unique or shared between protein groups (razor). (2) Each peptide was required to be quantifiable in at 50% of the biopsies in the meat- or no-meat eating group.

### Cell-free DNA analysis

cfDNA quantification using the Quant-iTTM PicoGreen® ds-DNA Assay Kit (Invitrogen) was performed using mouse plasma. By diluting Lambda DNA standard with TE-buffer (10 mM Tris–HCL, pH 7.5, 1 mM EDTA) a number of dilutions for a standard curve was created and loaded into a microplate. The samples were mixed with a constant amount of TE-buffer and loaded into the microplate. Triplicates were made of both standards and samples. The PicoGreen reagent was mixed with TE-buffer and kept in dark before it was added to the samples and standard DNA solutions. Then the microplate containing the samples was incubated 5 min at room temperature before analyzed in an EnSpire Multimode Plate Reader (PerkinElmer). The samples and standards were excited at 480 nm and the fluorescence emission was measured at 520 nm. A reagent blank without DNA is also performed. The value from the reagent blank is subtracted from the measurement from each of the wells containing DNA. A standard curve was calculated based on the measured fluorescence emission intensity and the known DNA concentration in the standards. Then the standard curve was used to calculate the concentration of DNA in the samples based on the measured intensities in the samples.

### Microbiome analysis by 16rRNA

#### DNA extraction

DNA was extracted using the QIAamp DNA stool mini kit automated on the QIAcube (Qiagen, Hilden, Germany). 1–2 stool pellets were ransferred to 0.70 mm Garnet Bead tubes (Dianova, Hamburg, Germany) filled with 1.1 mL ASL lysis buffer. Bead beating was performed using the a SpeedMill PLUS (Analytik Jena, Jena, Germany) for 45 s at 50 Hz. Samples were then heated to 95°C for 5 min with subsequent continuation of the manufacturer’s protocol.

#### Bacterial 16S rRNA gene sequencing and quality control

Variable regions V1 and V2 of the 16S rRNA gene were amplified using the primer pair 27F-338R in a dual-barcoding approach. PCR products were normalized using the SequalPrep Normalization Plate Kit (Thermo Fischer Scientific, Waltham, MA, United States), pooled equimolarily and sequenced on the Illumina MiSeq (Illumina Inc., San Diego, CA, United States). Demultiplexing after sequencing was based on 0 mismatches in the barcode sequences. Forward and reverse reads were merged using the Flash software, allowing an overlap of the reads between 250 and 300 bp. To eliminate low-quality sequences, the data were filtered by removing sequences with a sequence quality of less than 30 in less than 95% of the nucleotides. Chimeras were removed with UCHIME.

#### Microbiome bioinformatic analysis

Taxonomical classification was carried out using the RDP classifier, where classifications with low confidence at genus level (0.8) are organized in an arbitrary taxon of ‘unclassified_[family]’. For each sample, 10,000 sequences were randomly chosen to construct a taxon-by-sample abundance table. To infer functional capabilities from 16S rDNA amplicon sequencing data, reads were matched against the Greengenes OTU database (v13_5), down-sampled to 10,000 reads per sample, and used in the PICRUSt software. Resulting abundances were log2 transformed and subjected to hierarchical clustering (Perseus v1.6.0.2).

### Bioinformatics, statistics, and functional enrichment analysis

#### Differential proteomics analysis

Raw data in triplicate and label free quantitative (LFQ) values were assessed for normal distribution and all replicates with Person correlation below 0.95 removed prior to statistical filtering. To identify proteins with a statistically significant mean abundance change between group’s two-sided t-tests were performed. We applied permutation-based false positive control to correct for multiple hypothesis testing using standard parameters in Perseus v1.6.0.2 (S0 = 0.1, 250 randomizations, FDR < 5%). We statistically assessed the peptide abundance of Abcb1 a and b isoforms by Fishers exact test based on the iBAQ quantitative value.

#### Multivariate proteomics and data integration methods

We performed a sparse principal component analysis (sPCA) to that is an unsupervised model used to explore if the major source of variation in the proteomics datasets were related to each group of animals (Abcb1, FVB KO vs. WT) vs. timepoint (5w, 10w, 20w) vs. diet (Altromin, meat). Due to the small number of animals for week 5 and 30, we focused on the central time points: 10w and 20w. The input for the PCA was all quantified proteins and their abundances. Solemnly for the purpose of PCA, missing values (i.e., proteins where a quantitation value was not obtained for a given animal) were replaced by values from a normal distribution (width 0.3 and down shift 1.8), to simulate signals from low abundant proteins. The resulting data-table was imported into R (v 3.6.1) using PerseusR (v 0.3.4) in Rstudio (v 1.2.5001). The sPCA was made using the spca function contained in the MixOmics package (version 6.8.5). sparse Partial Least Squares-Discriminant Analysis model (sPLS-DA) was also adapted from MixOmics to identify the proteins with the highest predictive impact to identify the three treatment groups (37). The sPLS-DA is a supervised model that can identify the most variable proteins for classifying the treatment groups based on their proteome. We selected 20 proteins for the PCA model. The optimal number of components and variables on each component was determined by evaluating the performance of the model using the MixOmics perf and tune.splsda functions with a five-fold cross-validation and 50 repetitions. The best performance of the sPLS-DA model (lowest classification error rate) was achieved using two components with 10 and 35 proteins on components one and two, respectively. Scoreplots were made to illustrate if the model was able to cluster samples correctly based on the selected variables, and the cim function was used to create clustered image maps based on hierarchical clustering using the selected variables from the sPLS-DA. Finally, the network function was used to create a relevance network that shows negative and positive correlations between proteins and the three treatment groups greater than 0.6. This relevance network was imported into Cytoscape version 3.4.0 for graphical editing (38). Finally, a Data Integration Analysis for Biomarker discovery using Latent variable approaches for Omics studies (DIABLO) was adapted from the mixOmics R package to integrate colon proteomes with microbiota (39). This was to identify correlated colon proteins and bacteria that could simultaneously discriminate between the groups (WT CTL, WT MEAT, KO CTL and KO MEAT). The model had a design of 0.5. This means that the model balances between identifying biomarkers that are correlated across the two datasets and biomarkers that can discriminate between the four diet groups. The appropriate number of components was identified using the perf function with 3-folds cross-validation with 50 repeats, and the appropriate number of variables to keep on each component was identified using the tune function with 3-folds cross-validation and 160 repeats. Accompanying scoreplots, clustered image maps and circosplots were made to show how samples clustered based on the selected variables, and to visualize correlations between the selected variables.

#### Functional enrichment analysis

String-db was used to infer known and predicted protein–protein interactions and group regulated proteins (39). To identify underlying biological schemes in the list of significantly changed proteins, we performed a pathway enrichment analysis using the Reactome database in WebGestalt (40, 41). Pathways meeting FDR < 5% were considered statistically significantly enriched and are reported. We used all quantifiable proteins as background, to ensure sample-type specific pathways (e.g., proteins tagged as being extracellular in urine) would be subtracted. Functional enrichment analyzes based on Reactome Gene Sets, KEGG Pathways, GO Biological Processes, WikiPathways and CORUM, were also carried out in Metascape (42).

#### Cell-free DNA

For assessment of the cfDNA abundance before and after we performed Wilcoxon signed-rank test (R script).

#### Microbiome statistics

As most microbial abundance data did not have equal variances between the groups they were ranked before comparison. A general linear multifactorial ANOVA model was used with the random factor ‘KO’ and the fixed factor ‘Meat’ (Minitab 19, Coventry, United Kingdom). Subsequently, all value of ps were corrected by the Holm-Sidak False Discovery Rate (GraphPad Prism 8, San Diego, United States).

## Results

**Figure 1:**
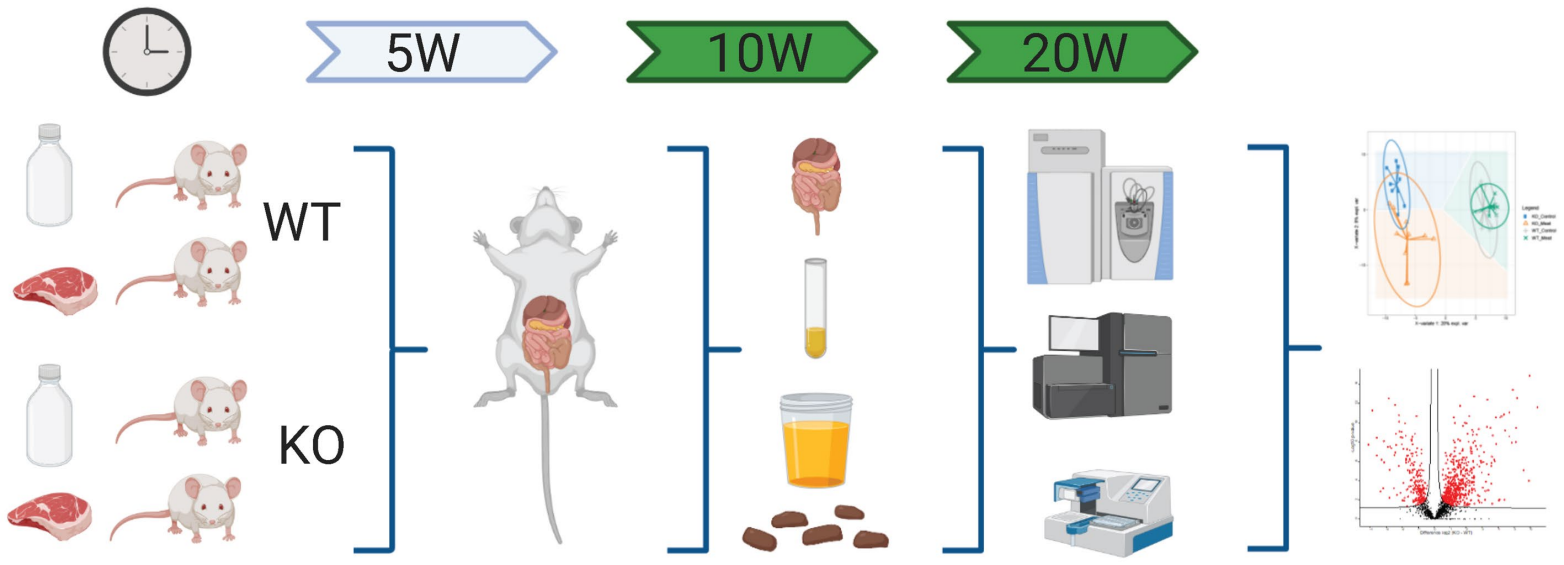
Schematic workflow of omics-based study investigating the impact of Abcb1a transporter deficiency and red meat containing diet in a time course of 5-20 weeks by multiomics correlated to microbiota. Created with BioRender.com.

To characterize the impact of the murine Abcb1a deficient mouse colitis model KO and WT mice were treated with diets based on either casein (CTL) or high-content of red meat (MEAT) and underlying molecular mechanisms related to Abcb1a function and impact of red meat-based diet were studied (Figure 1). We performed an unbiased hypothesis generating quantitative analyzes of the colon, ileum, and urine proteomes. The gut microbiota was profiled using bacterial amplicon sequencing based 16S RNA sequencing and correlated to genotype, diet and regional protein expression. Detailed information on mouse groups, diet, and proteomic data from each tissue, urine and microbiota, including the regulated pathways is provided in Supplementary Tables S1–S14.

### Lack of Abcb1a expression in colon induces a colitis phenotype

We investigated the macroscopic and microscopic changes in the colon at several time points (weeks 5, 10, and 20). Both examinations were performed blindly. The macroscopic appearance of the large intestine revealed the genetic status of the animals already after 5 weeks of the study. There was no macroscopically difference between the KO CTL and KO MEAT groups. The wall of the unopened colon of WT mice appeared, as expected, thin and transparent, with single fecal pellets visible through the wall. The epithelial surface of the longitudinally opened colon was unremarkable. In contrast, the KO mice’s colon was adherent to the caecum and small intestine and difficult to separate from the small intestine and mesentery. The wall of the KO colon was white, not transparent, thicker/edematous, tense upon touch/less elastic and shorter compared to WT animals. There was no fecal content in the lumen.

**Figure 2:**
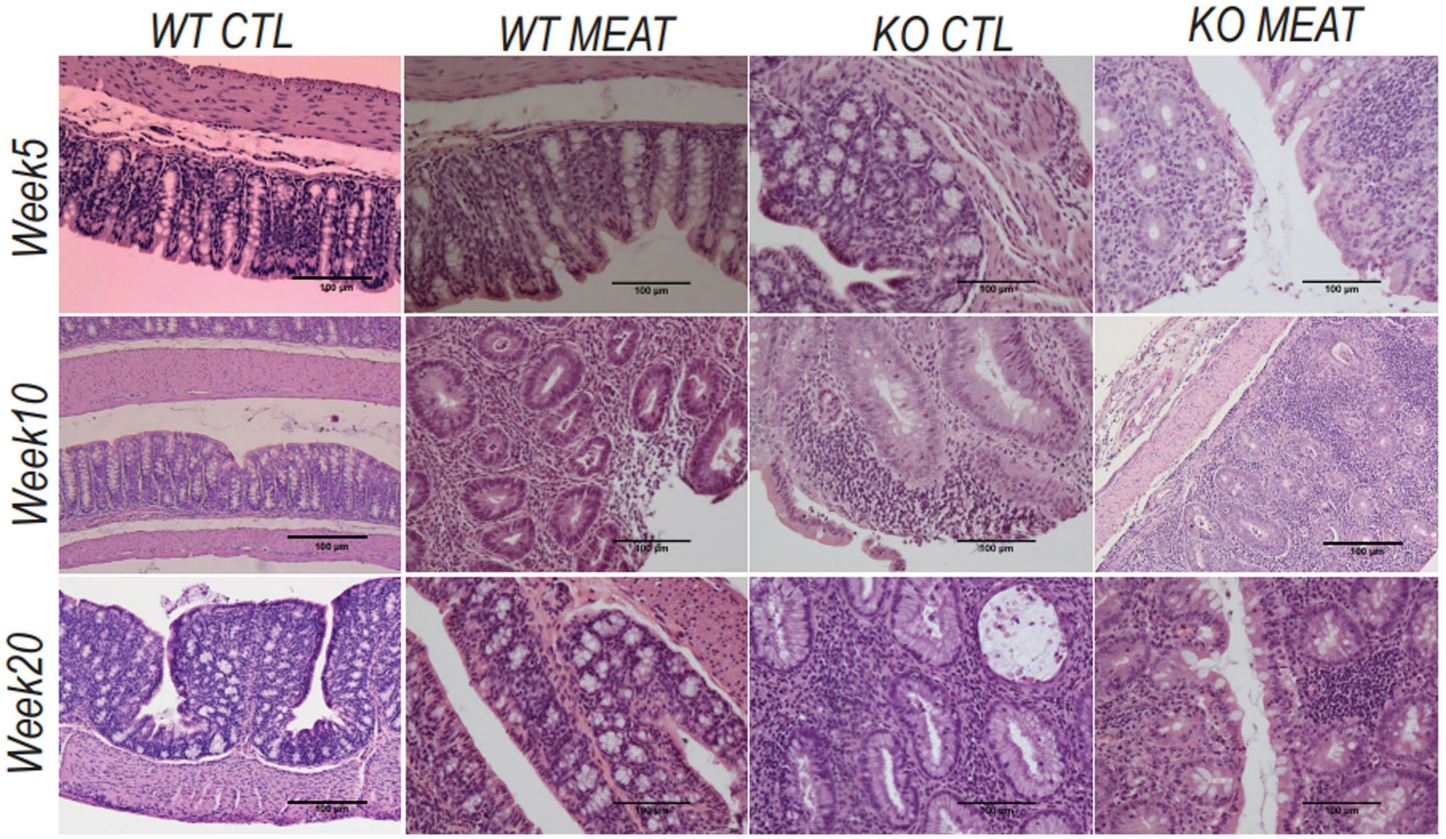
Lack of expression of Abcb1a in the colon induced colitis phenotype resulting in intestinal inflammation and tissue degradation. The progress in Abcb1a deficiency driven inflammation and impact of casein vs. meat diet on the colitis model are seen as alterations in tissue architecture (20x; scale bar 100µm) (KO Knockout; WT Wildtype).

Under microscopy, the WT groups showed normal histological morphology of the colon, while KO groups showed a colitis phenotype at the three time points evaluated. Crypt length in KO mice appeared to be increased compared to those in WT (Figure 2). We performed a standard histological examination, i.e., a descriptive evaluation, however, morphometry on crypts or mucosa was not planned in the study. In the colon of KO mice, a massive thickening of mucosa and inflammatory cell infiltrates reaching submucosa was seen. However, the two groups’ incidence or severity were not significantly different (Table 1). Other changes in the colon in both KO groups included cryptic abscesses (presence of neutrophilic polymorphs in crypt lumina), local mucosal erosions or ulcerations (small loss of epithelium with underlying inflammation), or ulcers (full mucosal thickness breaks with recognizable underlying inflammatory cell infiltration) but their incidences were low (Table 1).

**Table 1:** Type, incidence, and severity scores of histopathological changes in colons of Abcb1a knockout groups treated with either casein (CTL, control) or red meat-based diets (MEAT).

Parameter	Knockout CTL			Knockout MEAT		
Sacrifice	After 5 weeks	After 10 weeks	After 20 weeks	After 5 weeks	After 10 weeks	After 20 weeks
Thickening of colonic mucosa	2/2^a^	6/6	4/4	2/2	5/6	5/5^b^
Changes in epithelium						
Score E^c^						
Mean	1.5	1.8	1.3	2.0	1.2	1.4
Range	1–2	1–2	1–2	2–2	0–2	0–2
Inflammatory cell infiltration of colon						
Score I^d^						
Mean	3.5	2.3	2.8	3.0	1.7	2.6
Range	3–4	2–3	2–3	3–3	0–2	2–3
Total histologic score (mean E + mean I)	5.0	4.1	4.1	5.0	2.9	4.0
Mucosal erosion or ulceration	0/2	2/6	1/4	0/2	0/6	0/5
Ulcer	0/2	0/6	0/4	0/2	0/6	2/5
Cryptic abscess or cryptitis	1/2	2/6	2/4	2/2	3/6	5/5

^a^Incidence of a change is expressed by the number of colons with a given change of total number examined. ^b^Colon from one Knockout MEAT animal was not available for microscopic examination (Supplementary Table S2). ^c^Score system from Obermeier et al. (32) with modifications: 0: normal morphology; 1: loss of goblet cells; 2: loss of goblet cells in large area (in several folds); 3: loss of crypts; 4: loss of crypts in large area. ^d^Score system from Obermeier et al. (32) with modifications: 0: no infiltrate; 1: infiltrate around crypt basis; 2: infiltrate reaching muscularis mucosa; 3, loss of crypts; 4: infiltration of submucosa, tunica muscularis and serosa. (KO, Knockout; WT, Wildtype).

### Knock-out of Abcb1a gene alters the colon primarily and, to a lesser degree, ileum and urine proteomes and alterations are linked to gut microbiota interactions

**Figure 3:**
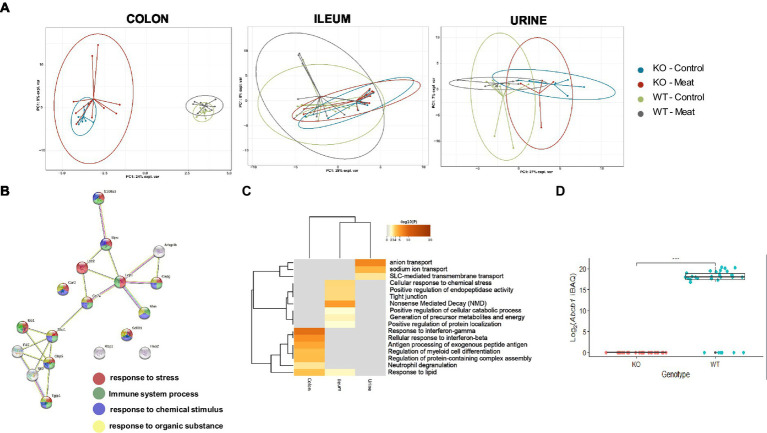
Comparison between WT and KO colon, ileum and urinary proteomes and relative expression of Abcb1a in the KO model. **(A)** Abcb1a knockout gave rise to the major source of variation in the colon proteome, resulting in clustering of mice corresponding to their genotype (KO vs. WT mice) in the sPCA model. A similar trend was observed in the ileum and urine proteomes, but the effect of Abcb1a knockout did not give rise to as much variation in herein to discriminate between WT and KO mice as evidenced by overlap between 95% confidence ellipses. **(B, C)** Functional enrichment of colon proteins explaining the difference between WT and KO were associated with immune-related processes, transport of organic substances, neutrophil degranulation and cellular stress. Across proteome comparison of enriched biological processes, we identify processes related to metabolism and immune-associated processes. **(D)** Validation of expression of Abcb1a transporter in the colon of WT and Abcb1a KO mice (***: Fisher’s exact test *p*-value < 0.001). (KO Knockout; WT Wildtype).

Having observed the macroscopic and histological changes in the colon between the four animal groups, we proceeded with proteomic assessment of the Abcb1a KO induced changes in the colon, ileum and urine assessment of the proteomic alterations by the diet. Initially, we focused investigations on the impact of diet at the onset of the intestinal colitis phenotype (weeks 10 and 20; Supplementary Table S2). No significant differences were detected comparing the week 10 and week 20 samples, as determined by a PCA and a differential analysis with two-sample t-tests (adjusted *p* > 0.05; data not shown). Therefore, we treated the two time points as one. In the colon samples, the genetic background could describe the main component 1 variation in the datasets, as displayed by the sPCA that simplify the genetic model and isolate variables affected by the diet and genetic model (Figure 3A). However, this was not the case for the ileum and urine samples where no clear sample groupings were found, indicating a relatively higher impact of Abcb1a KO in the colon, in agreement with this being the only intestinal location of Abcb1a expression (43). Hence, the relative impact of diet seemed insignificant.

We further investigated the biological meaning of the discriminative proteins on component1 (20 most discriminative proteins), explaining the difference between WT and KO mice using STRING and Metascape functional enrichment analysis (Figures 3B,C). The analysis revealed enrichment of biological processes likely related innate immune response and response to cellular stress associated with the KO phenotype including neutrophil degranulation. Among the affected expressed proteins in colon, multiple proteins were associated with IBD (44) and IFN-gamma-dependent regulatory circuits in immune inflammation (45). Other GO categories were affected in the ileum, whereas affected biological processes in the urine proteome were associated with anion transport and proteolysis.

We further validated the distinct endogenous protein expression level and identity of murine Abcb1 isoforms by positive protein identification of Abcb1a or Abcb1b in 0/30 KO mice and 25/31 WT (Figure 3D), demonstrating a successful KO of Abcb1a (Fishers exact test value of *p* <10^−5^). Although Abcb1a and Abcb1b comprises 40 and 38 protein-unique tryptic peptides, our identification of Abcb1 relied on one high confidence (FDR < 1%) unique peptide, and we were unable to specify the Abcb1 subfamily. This could indicate a relatively low Abcb1-expression, in agreement with the lack of identification in six of the WT mice where the abundance likely dropped below the detection limit. Therefore, to confirm the expression of Abcb1a rather than Abcb1b in the WT mice, we performed an extended highly sensitive proteome analysis for higher sequence coverage of eight representative colon samples. As a result, Abcb1 was unambiguously identified in 0/4 KO colon samples and 4/4 WT samples with 5 high confidence peptides (FDR < 1%), four of which were unique to Abcb1a, and the one peptide shared between Abcb1a and Abcb1b. Hence, we confirmed Abcb1a expression in WT mice with high confidence and tissue absence below the detection limit of both isoforms in KO mice.

### Proteomics suggest that Abcb1a inactivation induces activation of the immune system

**Figure 4:**
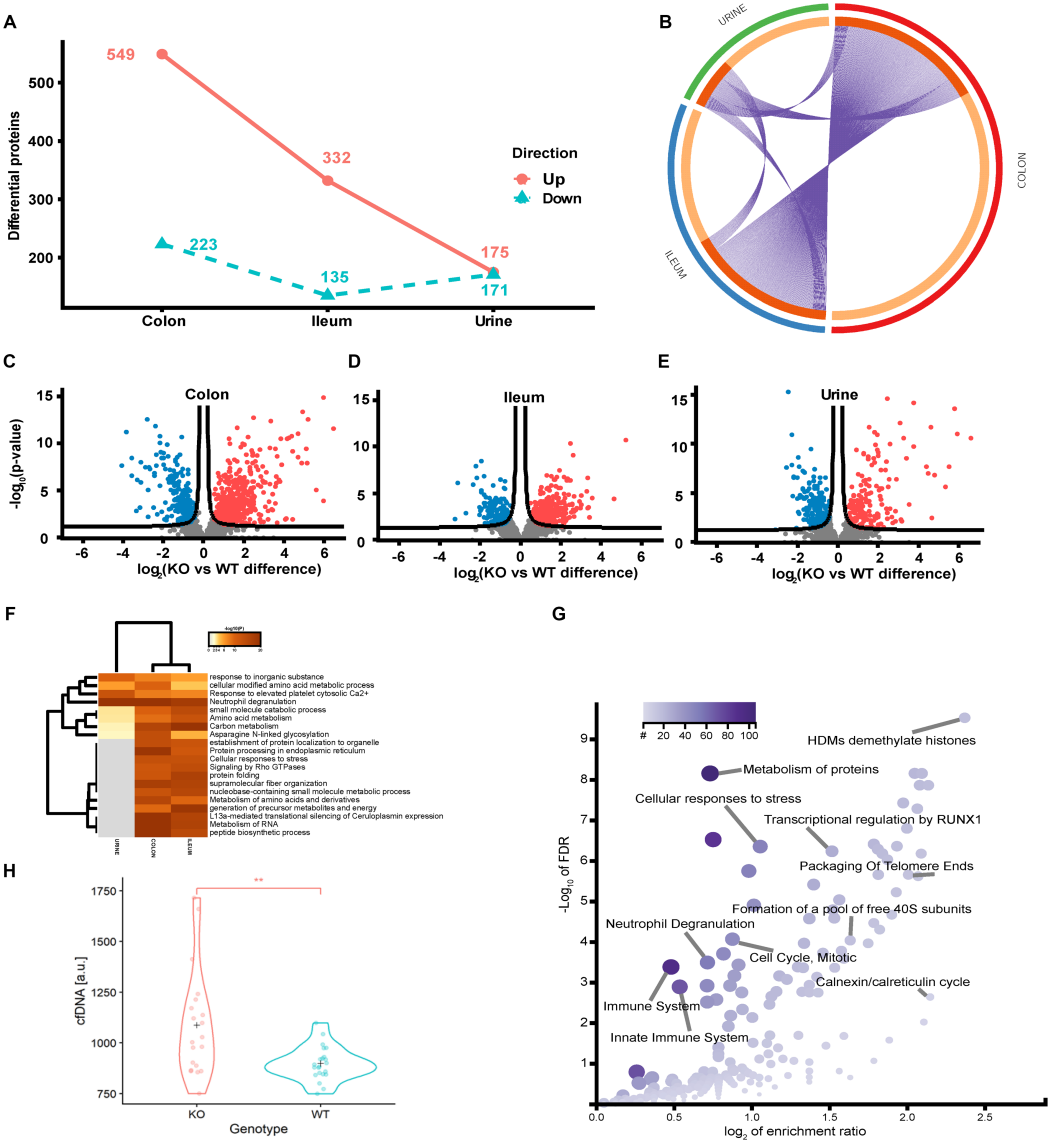
Quantitative proteome analysis of the Abcb1 KO model and comparison of the colon, ileum and urinary proteomes. **(A)** Summary of the significantly up and down regulated protein within the analyzed proteomes. **(B)** Overlap of groups in the distinct proteomes. Dark orange color represents the proteins that appear in multiple lists and light orange color represents proteins that are unique to colon, ileum or urine. Purple lines link the protein shared by multiple lists. **(C-E)** Quantitative comparisons (log-fold-change) based on multiple-hypothesis testing indicating the number of specific proteins that were uniquely regulated within the two tissues colon, ileum and urine significant proteins of the colon, ileum, and urinary samples. **(F)** Statistically enriched terms (GO/KEGG, canonical pathways, hall mark genes) across the three proteomes. **(G)** scatterplot of enriched GO Biological process groups with indication of relevant processes. **(H)** Increase in plasma cell free DNA by NETs associated neutrophil degranulation (*p*-value = 0.002).

To identify the regulated protein and cellular processes affected by functional loss of transporter activity and the impact of diet on the protein level, we performed a differential analysis independent of diet, comparing the KO and WT samples for each sample type separately. In the colon, ileum and urine, a total of 772 (26.1%), 467 (14.8%) and 346 (25.3%) differentially regulated proteins (*p* < 0.05 FDR) were identified (Figures 4A–E). The colon is the main site of Abcb1a biosynthesis, and it was at this site that we observed the greatest impact on the proteome. Each core proteome contained a degree of overlap, including a fraction secreted in urine (Figure 4B). Neutrophil degranulation is an highly enriched process as observed in colon and ileum as well as urine (Figures 4F,G). While the ileum and colon proteomes reflect local effects limited to these locations, urine is a systemic body fluid representing elements from the entire body (46). Bacterial translocation over the mucosa may activate NETs in the gastrointestinal and urinary tract. We have previously identified neutrophil degranulation activated as a central antimicrobial defense mechanism in the gut intestinal barrier (47). Thus, we investigated if elevated cfDNA in the plasma from the two groups of animals could be correlated with Abcb1a genotype status and degree of inflammation. We determined the plasma level of cfDNA as a pseudo indicator for putative systemic NETs activation because neutrophil degranulation-related proteins were found in the colon, ileum and urine (48). The systemic level of cfDNA level is significantly elevated in the KO group (Figure 4H; value of *p* = 0.002).

To further identify underlying biological schemes associated with the Abcb1a KO phenotype in the significant lists of differentiated proteins, we performed separate Reactome pathway enrichment analyzes for the up- and down differentiated proteins for the three samples (Table 2).

**Table 2:** Enriched reactome pathways of significantly regulated proteins comparing Abcb1 knockout to wildtype mice, for colon, ileum, and urine, respectively.

KO upregulated reactome pathways	Pathway description	**O**^**a**^	**value of p**^**b**^	**FDR**^**c**^
Urine				
R-MMU-168256	Immune System	11	2.26e-02	0.57
R-MMU-6798695	Neutrophil degranulation	11	9.13E-02	1
Ileum				
R-MMU-72689	Formation of a pool of free 40S subunits	14	8.07E-05	3.00E-02
R-MMU-72766	Translation	17	9.60E-05	3.00E-02
Colon				
R-MMU-72766	Translation	35	5.39E-11	2.59E-08
R-MMU-72689	Formation of a pool of free 40S subunits	29	8.52E-11	2.59E-08
R-MMU-156827	L13a-mediated translational silencing of Ceruloplasmin expression	30	4.22E-10	7.98E-08
R-MMU-392499	Metabolism of proteins	86	5.25E-10	7.98E-08
R-MMU-72706	GTP hydrolysis and joining of the 60S ribosomal subunit	30	1.50E-09	1.83E-07
R-MMU-72613	Eukaryotic Translation Initiation	30	2.74E-09	2.38E-07
R-MMU-72737	Cap-dependent Translation Initiation	30	2.74E-09	2.38E-07
R-MMU-72695	Formation of the ternary complex, and subsequently, the 43S complex	20	5.29E-09	4.02E-07
R-MMU-975956	Nonsense Mediated Decay (NMD) independent of the Exon Junction Complex (EJC)	24	9.40E-09	6.35E-07
R-MMU-72649	Translation initiation complex formation	21	1.92E-08	1.06E-06
R-MMU-72662	Activation of the mRNA upon binding of the cap-binding complex and eIFs, and subsequent binding to 43S	21	1.92E-08	1.06E-06
R-MMU-72702	Ribosomal scanning and start codon recognition	21	4.16E-08	2.11E-06
R-MMU-1799339	SRP-dependent cotranslational protein targeting to membrane	22	5.20E-08	2.43E-06
R-MMU-927802	Nonsense-Mediated Decay (NMD)	24	1.24E-07	5.01E-06
R-MMU-975957	Nonsense Mediated Decay (NMD) enhanced by the Exon Junction Complex (EJC)	24	1.24E-07	5.01E-06
R-MMU-74160	Gene expression	74	3.58E-06	1.36E-04
R-MMU-6798695	Neutrophil degranulation	53	2.34E-05	8.37E-04
R-MMU-446203	Asparagine N-linked glycosylation	30	1.56E-04	5.26E-03
R-MMU-6811434	COPI-dependent Golgi-to-ER retrograde traffic	11	2.01E-04	6.44E-03
R-MMU-901042	Calnexin/calreticulin cycle	6	2.93E-04	8.89E-03
R-MMU-168249	Innate Immune System	70	4.73E-04	1.35E-02
R-MMU-8856688	Golgi-to-ER retrograde transport	15	4.88E-04	1.35E-02
R-MMU-168256	Immune System	84	6.20E-04	1.64E-02
R-MMU-199977	ER to Golgi Anterograde Transport	19	1.36E-03	3.43E-02
R-MMU-6807878	COPI-mediated anterograde transport	15	1.49E-03	3.62E-02
Urine				
N/A				
Illum				
R-MMU-1430728	Metabolism	39	2.41E-05	1.50E-02
Colon				
R-MMU-1430728	Metabolism	60	8.55E-07	5.20E-04
R-MMU-1428517	The citric acid (TCA) cycle and respiratory electron transport	16	2.73E-05	8.29E-03

^a^O: Number of observed significant proteins in the pathway. ^b^pathway enrichment value of p. ^c^pathway enrichment false discovery rate (FDR) calculated in the relevant protein background.

In accordance with the tissue-specific expression of Abcb1 in the colon, we identified the highest number of affected pathways in these samples. The main impact of Abcb1 KO was an increased protein synthesis and pathway upregulation, as demonstrated in the increased number of pathways for these colon proteins and the upregulation of translation pathways in the colon and ileum samples.

In Abcb1 KO mice, a shift toward protein metabolism, reduced carbon metabolism, and immune system activation were observed. In urine, ileum, and colon, we observed pathways related to increased immune activation and protein synthesis and only reduced metabolism in the ileum and colon. The urine reflects the systemic response to a certain extent. Most proteomic alterations in the colon agreed with the expected Abcb1a expression in this tissue. However, the KO phenotype was also observed in ileum and urine proteomes. Therefore, we explored discriminative features of colon, ileum and urine proteomes to investigate the minimal impact of diet and systemic functionality of the efflux transporter. We investigated if the sPLS-DA, as a supervised model, could identify proteins reflecting diet-associated effects in the colon, ileum, and urine.

**Figure 5:**
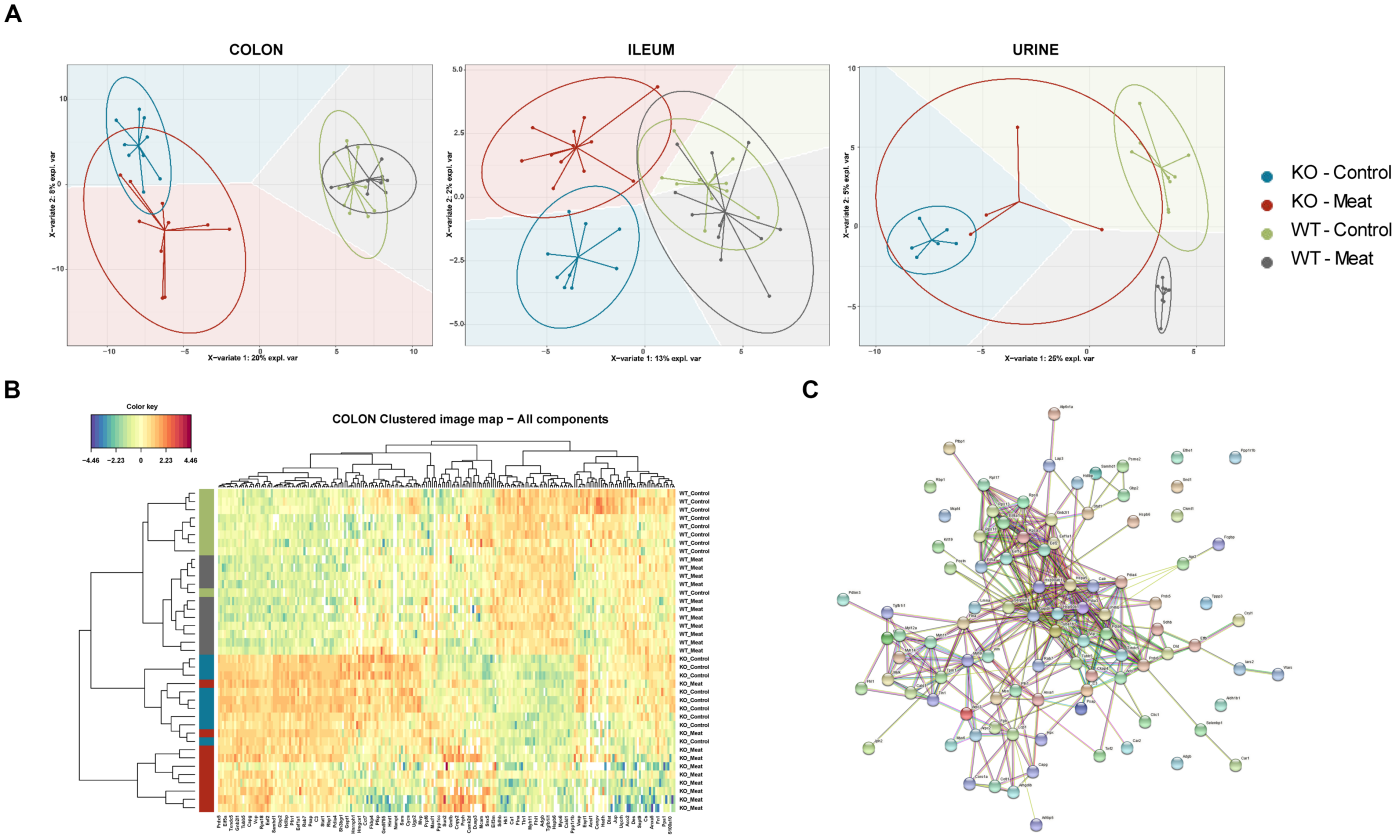
Regional Abcb1 expression in the KO model and comparison of the colon, ileum and urinary samples shows distinct proteome profiles. **(A)** A supervised model, sparse Partial Least Squares-Discriminant Analysis (sPLS-DA) was used to identify discriminative proteins in the proteomes of the four animal groups. Colored background indicates the computed prediction background for predicting class of samples. **(B)** Hierarchical clustering of regulated proteins in colon reveals the most important variables for discriminating between the groups, showed a clustering of all four groups. **(C)** Protein–protein interaction network analysis of discriminative proteins between wildtype and knockout groups by STRING.

The sPLS-DA scores plots visualize the ability of the models to discriminate between samples based on genotype and diet, and the colored background represents the prediction areas, that is, how the sPLS-DA models predict the class of unknown samples (Figure 5A). As expected, a clear discrimination between genotypes was observed on component 1 within all proteomes, and to a lesser degree, a discrimination between diet groups was observed on the remaining components.

The selected variables on each component were used to create heatmaps based on hierarchical clustering. A combined heatmap using all the variables for discriminating between colon samples on components 1–3 showed four clusters that roughly correspond to the four groups (Figure 5B). The core of discriminative proteins in the Abcb1a deficient mice was associated with processes related to immune regulatory processes and associated with IBD (Figure 5C) (44).

### The Abcb1a KO mice showed major differences in microbiota composition compared to WT mice, while feeding red meat only induced minor differences

The main expression of Abcb1a in the colon and the observed impact prompted us to investigate the impact of Abcb1a KO on the gut microbiota predominant in the colon.

**Figure 6:**
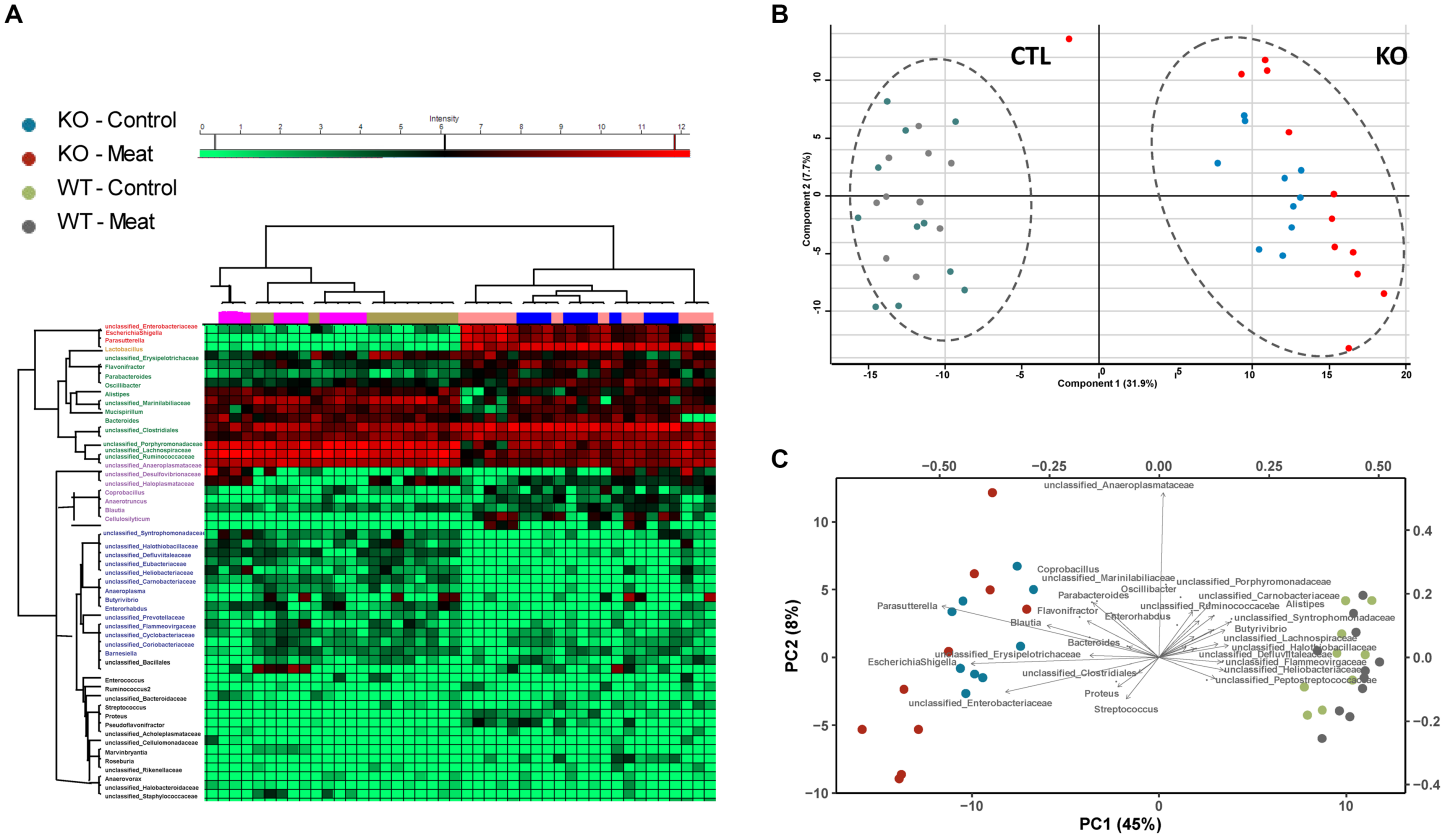
Microbiota community profiling by 16S rRNA gene sequencing. **(A)** Cluster analysis of the most abundant strains groups using unsupervised cluster analysis; **(B)** Principal component analysis of strain abundances enabling clustering of WT and KO animals and association of strains to the genotype. **(C)** Biplot indicating differences in microbiota enabling association of closely related abundance alterations between bacterial strains.

The overall clustering of the microbiota did not differ in relation to the intake of red meat, while microbiota profiles of WT and KO mice were significantly different, as determined by an unsupervised PCA (Figures 6A–C). The KO mice had an increased abundance of bacteria within Proteobacteria, i.e., well classified *Eschericia/Shigella* spp. and *Parasutterella* spp., as well as some unclassified Enterobacteriaceae spp. Also, genera within Firmicutes, i.e., *Blautia* spp., *Cellulosilyticum* spp., *Coprobacillus* spp., and *Flavonifractor* spp. increased their abundance in the KO mice. The abundance of *Bacteroides* (Bacteroidetes) was slightly but significantly increased in the KO mice. Some well classified genera within Firmicutes, i.e., *Oscillobacter* spp. and *Butyrovibrio* spp. and some less well-classified families within Firmicutes, i.e., Ruminococcaceae, Lachnospiraceae, Carnobacteriaceae, Peptostreptococcaceae, Heliobacteriaceae, and Defluviitaleaceae, were decreased in abundance in the KO mice. *Alistipes* spp. (Bacteroidetes) were decreased in abundance in the KO mice, and so were some less classified Bacteroidetes families, i.e., Porphyromonadaceae and Flammeovirgaceae. The family Halothiobacillacea (Proteobacteria) was significantly decreased in abundance in the KO mice. A significant increase in the abundances of *Lactobacillus* spp. (Firmicutes), *Blautia* spp. and *Barnesiella* spp. (Bacteroidetes) in the mice fed red meat (Table 3).

**Table 3:** Abundances of bacteria significantly differ between WT and Abcb1a KO mice (light gray) and mice fed either a casein-based or a red meat-based diet (Dark gray).

	Knockout			Wild-type			Meat			Plant			Knock-out/Wild-type %	Meat/Plant %	GLM ANOVA		Holm-Sidak	
	Mean ± SD			Mean ± SD			Mean ± SD			Mean ± SD					Value of *p*		Adjusted value of *p*	
	*N* = 22			*N* = 24			*N* = 20			*N* = 26					Geno-type	Diet	Geno-type	Diet
*Blautia*	4.9	±	3.0	0.0	±	0.0	1.6	±	2.0	2.9	±	3.8	NA	53.7	0.000	0.000	0.000	0.000
*Cellulosilyticum*	2.0	±	3.1	0.0	±	0.0	0.0	±	0.0	1.7	±	3.0	NA	0.0	0.000	0.000	0.000	0.000
*Escherichia/Shigella*	8.7	±	1.7	0.1	±	0.4	5.3	±	4.4	3.4	±	4.4	5793.2	154.2	0.000	0.520	0.000	1.000
*Parasutterella*	10.8	±	0.7	0.3	±	0.6	6.8	±	5.4	4.2	±	5.1	3339.3	163.7	0.000	0.013	0.000	0.351
*Coprobacillus*	3.5	±	2.1	0.4	±	1.0	1.5	±	2.1	2.1	±	2.3	879.9	73.4	0.000	0.012	0.000	0.337
unclass._Enterobacteriaceae	8.7	±	1.6	1.7	±	1.8	5.9	±	3.8	4.4	±	4.0	514.4	133.3	0.000	0.778	0.000	1.000
*Flavonifractor*	5.9	±	1.4	2.7	±	1.1	4.2	±	2.2	4.3	±	2.0	219.7	99.2	0.000	0.051	0.000	0.744
unclass._Haloplasmataceae	3.3	±	2.2	1.5	±	1.6	2.7	±	2.2	2.1	±	2.0	216.3	125.5	0.006	0.882	0.075	1.000
unclass._Erysipelotrichacea	7.7	±	1.4	4.3	±	1.3	6.8	±	2.5	5.2	±	1.5	176.6	129.9	0.000	0.056	0.000	0.763
*Parabacteroides*	7.6	±	1.4	5.3	±	0.9	6.6	±	1.4	6.3	±	1.8	143.0	105.5	0.000	0.967	0.000	1.000
*Anaerotruncus*	3.8	±	1.5	3.0	±	1.3	3.1	±	1.6	3.5	±	1.3	125.6	89.7	0.034	0.383	0.268	1.000
unclass._Desulfovibrionacea	4.4	±	1.9	3.6	±	3.7	3.9	±	2.8	4.0	±	3.1	122.7	96.8	0.947	0.703	0.989	1.000
*Barnesiella*	1.9	±	1.7	1.6	±	1.4	2.5	±	1.4	1.1	±	1.4	121.0	237.9	0.841	0.001	0.989	0.034
*Bacteroides*	10.9	±	0.7	9.5	±	1.0	10.2	±	0.8	10.1	±	1.3	115.1	101.5	0.000	0.119	0.000	0.946
unclass._Clostridiales	9.3	±	0.6	8.4	±	0.8	9.2	±	0.8	8.6	±	0.8	111.6	106.5	0.000	0.043	0.000	0.720
*Lactobacillus*	6.6	±	1.7	6.7	±	2.2	7.7	±	1.6	5.8	±	1.7	98.2	133.6	0.270	0.000	0.890	0.000
unclass._Anaeroplasmataceae	2.8	±	3.5	2.9	±	3.6	3.3	±	3.7	2.5	±	3.4	96.6	130.9	0.729	0.466	0.989	1.000
unclass._Marinilabiliaceae	6.6	±	1.9	7.7	±	1.3	7.3	±	1.4	7.1	±	1.9	86.3	102.4	0.009	0.388	0.103	1.000
*Mucispirillum*	6.6	±	2.9	7.6	±	1.1	6.5	±	3.0	7.6	±	1.2	86.0	86.3	0.673	0.631	0.989	1.000
unclass._Ruminococcaceae	8.4	±	1.2	10.1	±	0.4	8.8	±	1.5	9.6	±	0.9	83.7	92.0	0.000	0.102	0.000	0.924
unclass._Porphyromonadaceae	9.0	±	1.9	11.2	±	0.5	9.9	±	1.8	10.3	±	1.7	80.3	96.4	0.000	0.996	0.000	1.000
unclass._Lachnospiraceae	8.6	±	1.0	11.2	±	0.5	9.4	±	1.7	10.4	±	1.3	76.8	89.7	0.000	0.028	0.000	0.585
unclass._Cyclobacteriaceae	1.7	±	1.4	2.2	±	1.6	1.8	±	1.4	2.1	±	1.6	76.7	86.7	0.313	0.788	0.895	1.000
*Oscillibacter*	5.7	±	2.0	7.6	±	1.0	6.1	±	2.1	7.1	±	1.5	74.5	86.4	0.000	0.307	0.000	0.999
*Alistipes*	6.4	±	2.7	9.4	±	1.2	7.6	±	3.1	8.3	±	2.0	68.5	91.6	0.000	0.519	0.000	1.000
*Anaeroplasma*	1.7	±	3.2	2.6	±	3.5	2.1	±	3.6	2.2	±	3.2	64.2	94.6	0.338	0.885	0.895	1.000
unclass._Coriobacteriaceae	1.9	±	1.5	3.0	±	1.2	2.5	±	1.4	2.3	±	1.5	62.8	108.7	0.010	0.311	0.105	0.999
unclass._Flammeovirgaceae	1.4	±	1.2	2.7	±	1.2	1.9	±	1.4	2.2	±	1.4	49.6	88.5	0.000	0.820	0.000	1.000
unclass._Eubacteriaceae	1.2	±	1.8	2.5	±	2.0	1.7	±	2.0	2.0	±	2.0	49.0	85.8	0.015	0.990	0.140	1.000
unclass._Prevotellaceae	0.9	±	1.2	2.3	±	2.3	1.6	±	1.8	1.6	±	2.1	41.3	98.1	0.056	0.711	0.369	1.000
unclass._Syntrophomonadacea	1.2	±	1.8	3.4	±	1.7	1.9	±	1.8	2.7	±	2.2	36.0	71.2	0.000	0.605	0.000	1.000
*Enterorhabdus*	0.5	±	0.8	1.6	±	1.2	0.7	±	0.9	1.4	±	1.2	33.0	54.7	0.002	0.230	0.028	0.997
unclass._Carnobacteriaceae	0.5	±	1.0	2.1	±	1.4	1.6	±	1.7	1.1	±	1.2	22.5	141.5	0.000	0.045	0.000	0.725
unclass._Halothiobacillacea	0.8	±	1.1	3.7	±	1.6	2.2	±	1.9	2.4	±	2.1	20.7	91.9	0.000	0.280	0.000	0.999
*Butyrivibrio*	0.7	±	1.3	3.5	±	2.0	1.9	±	2.1	2.3	±	2.4	18.6	81.6	0.000	0.570	0.000	1.000
unclass._Peptostreptococcac	0.3	±	1.4	3.1	±	2.2	2.2	±	2.9	1.5	±	1.8	9.9	148.4	0.000	0.046	0.000	0.725
unclass._Heliobacteriaceae	0.2	±	1.0	3.1	±	1.4	1.8	±	2.1	1.6	±	1.7	6.9	116.6	0.000	0.021	0.000	0.493
unclass._Defluviitaleaceae	0.1	±	0.6	2.6	±	1.6	1.6	±	2.1	1.2	±	1.4	5.0	130.8	0.000	0.029	0.000	0.586

White row show bacteria only significantly different before Holm-Sidak correction.

**Figure 7:**
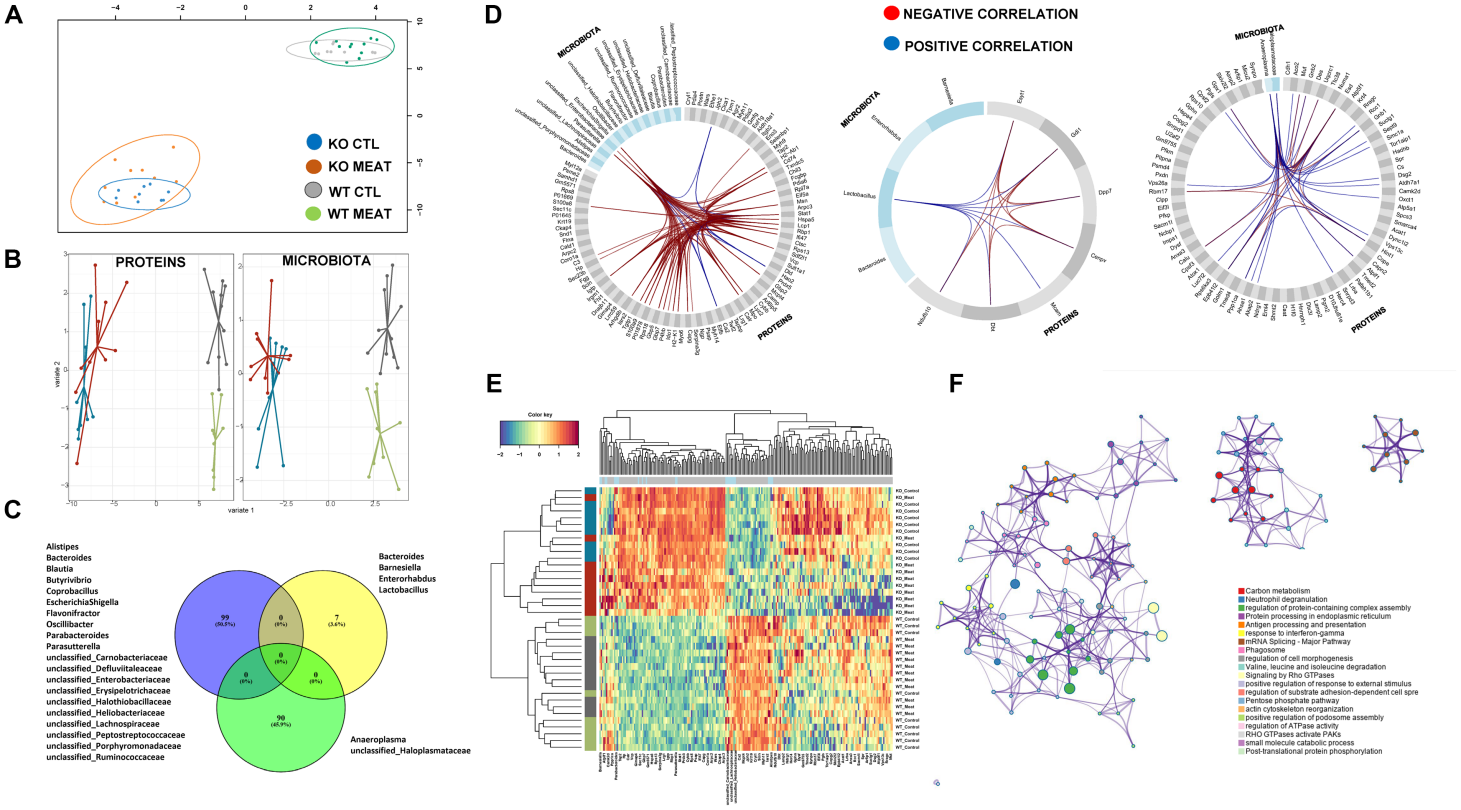
Microbiota community and affected protein expression in colon. **(A)** Correlation between component1 of colon proteome and microbiota. **(B)** DIABLO sample plots for colon protein block (left) and microbiota block (right) showing discrimination between WT vs. KO groups on component1. **(C)** Microbiota and overlap of proteins in correlations components01-03. **(D)** Circosplots showing correlation between variables from component 1 to 3. **(E)** Heatmap of all the protein variables selected on component 1 to 3 showing a clear clustering based on genotype, and to a lesser degree, clustering based on diet. **(F)** Protein functional association of 99 proteins in DIABLO component 1 to 3 with associations to innate immune response and response to chemical stress.

The striking microbiota community difference led us to investigate if proteomic alterations were specific to CTL and KO groups. We employed an integrative approach for multiple data sets in a supervised analysis using DIABLO. Specifically, DIABLO was used to identify highly correlated variables within the colon proteome and microbiome that can simultaneously discriminate between animals based on genotype (WT, KO) and diet with high specificity (Figures 7A,B). The DIABLO analysis had a total of three components. Component 1 within the microbiota block consisted of 20 microbiota strains, three (*Parasutterella*; *unclassified_Enterobacteriaceae*; *Escherichia Shigella*) of which were highly correlated (cut-off 0.9) to 26 out of the 99 protein variables selected on component 1 within the colon proteome block (Figures 7C,D), inflammatory mechanisms, including neutrophil degranulation (Figure 7F). Functional enrichment analysis revealed that 17 out of 26 proteins were related to neutrophil degranulation (value of *p* 1.1E-10), including C3, CAMP, CTSC, CYBB, H2- K1, HP, ITGB2, LYZ2, MPO, PSAP, S100A8, S100A9, SERPINA3G, GMFG, TXNDC5, CKAP4, VCP, CHIL3, IDO1 and GBP5. Four variables were selected on component 2 within the microbiota block, hereof, *Lactobacillus* was correlated (cut-off 0.5) to the seven proteins selected on component 2 within the colon protein block. Finally, two variables were selected on component 3 within the microbiota block (*Anaeroplasma* and *unclassified_Haloplasmataceae*). These two strains were correlated (cut-off 0.5) to 26 of the 90 variables selected on component 3 within the colon protein block. Cluster analysis confirmed a distinct association abundance of specific strains and colon proteins based on the genetic background of the animals and, to a lesser degree, diet (Figure 7E), whereas the functional role includes inflammatory mechanisms, including neutrophil degranulation.

## Discussion

We hypothesized that gut microbiota and gastrointestinal inflammation would significantly differ between WT and KO mice and would be influenced by diet supplementation. Diet composition is a contributing factor to IBD (49, 50). A large cohort study reported that protein intake was associated with an increased risk of incident IBD (16). Animal protein has been suggested to contribute to gut inflammation in IBD (51). Our study suggests no major differences between the origin of protein from meat or casein (Figure 3A). Analysis of the Abcb1 protein expression level of mouse gene variants confirmed no Abcb1a or Abcb1b in the colon, while gross and microscopic examinations also confirmed the colitis phenotype, in all KO mice. Surprisingly, there was no significant interaction between Abcb1a status and meat intake, suggesting that the contribution of a red meat-based protein source might be too small, in relation to a complete lack of the Abcb1 transporter in the animal model. The role of dietary macronutrients in the etiology of IBD have previously been investigated, and concluded that the high consumption of meat or fish but not of eggs or dairy products was associated with IBD risk (52). Another study indicates that dietary red meat aggravated the severity of colitis based on clinical signs of disease in an animal model, emphasizing the potential of diet to modulate mucosal (53).

Our proteomics analysis of the colon, ileum and urine shows that Abcb1 KO mice activate the immune system in relation to neutrophil degranulation and small-molecule stress as well as a shift toward protein metabolism on the cost of carbon metabolism. The latter was supported by essential changes in the gut microbiota composition of the KO mice compared to the WT mice (54). We have previously reported the formation of NETs being part of neutrophil degranulation in human ulcerative colitis mucosal biopsies (55). We found neutrophil degranulation in the colon samples, a previously reported site of NETs formation in inflammatory diseases and in the ileum and urine (47). Neutrophil degranulation is associated with NETs formation and the loss of barrier integrity in inflamed intestinal tissues (56). Such granules are composed of unfolded DNA treads coated with cytosolic and granular antimicrobial enzymes found previously in biofluids, intestinal tissue as well as the urine tract (16, 18). They may be an early marker and essential defense mechanism in IBD, where neutrophils migrate to form crypt abscesses, a hallmark of IBD (19).Tissue-associated NETs formation by neutrophil degranulation has not been described in the ileum. However, we found neutrophil degranulation more pronounced in the colon samples compared to the ileum samples (STRING analysis value of p 4.10e-25 vs. 2.76e-13). In our study, we found an elevated level of cfDNA in the mouse plasma to support the systemic and biofluid-associated effect of neutrophil degranulation. This free DNA finding in biofluids is reported in other autoimmune disorders and bacterial infections (48, 57, 58). cfDNA is implicated in managing IBD not only as biomarkers distinguishing patients from healthy people and differentiating active from inactive disease state, but also as a potential therapeutic target (59).

In the KO mice, the increased abundance of some Proteobacteria, especially *Eschericia*/*Shigella* is in accordance with previous DGGE and qPCR based observations (60). An important microbial-associated molecular pattern (MAMP) from Proteobacteria, and especially from *Eschericia coli* is lipopolysaccharides (LPS), which activate the Toll-like receptor 4 (TLR4). TLRs have been found to impact many aspects of IBD etiology, including immune responses, microbiota (61) and TLR polymorphisms linked to IBD (61). Indeed, the capacity of foodstuffs to induce innate immune activation of human monocytes *in vitro* was found to be dependent on the food content of stimulants of TLR2 and TLR4 (13). Through the NFκβ and p38 MAP kinase pathways, LPS stimulation will induce secretion of TNF-α and other innate cytokines (62). The colitis in Abcb1 KO mouse as described by Panwala and colleagues in 1998 (11) is characterized by high levels of the cytokines INF-γ, TNF-α, IL-1β, IL-6 and IL-17, thus resembling the findings in UC patients (63). Therefore, LPS from Proteobacteria or other gram-negative bacteria may be essential for high levels of cytokines reported. Another Proteobacterium, *Parasutterella*, which we observed with increased abundance in the Abcb1 KO mice is also known to have an increased abundance in the gut of patients another form of IBD (64). Also, another study has associated an increased abundance of Parasutterella with dysbiosis, or a lack of diversity in the gut microbial composition. This is correlated to intestinal dysfunction, such as IBD. Hallothiobacillaceae, which also belong to Proteobacteria, and which were significantly decreased in our KO mice, have previously been shown to vanish when IL-10 KO mice, one of the most commonly used IBD models, have gut inflammation induced with *Atopobium parvulum* (65). The relative abundances between members of the class Clostridia differed between KO and WT mice, which makes sense in relation to the observed changes in tissue metabolism. Lachnospiraceae is a family of anaerobic, spore-forming Clostridiales known for their ability to ferment plant polysaccharides into short-chain fatty acids, such as butyrate and acetate (66). Some well-defined Lachnospiraceae spp. were increased in abundance in KO. These included *Blautia* spp., which in the horse gut correlates to the expression of IL-10 (67). Also, the closely related *Cellulosilyticum* spp. were in increased abundance in KO mice. These are species living in environments with complex carbohydrate structures, such as the rumen of ruminants (68) and river beds (69). *Cellulosilyticum* spp. have been associated with IBD for patients having a higher perceived stress level (14). Other members of the class Clostridia were decreased in abundance in the KO mice, e.g., *Butyrovibrio* spp., which are known to metabolize carbohydrates and fatty acids, e.g., in the rumen of ruminants (70), as well as some unclassified Heliobacteriaceae, Lachnospiraceae, Peptostreptococcaceae, Defluviitaleaceae, Heliobacteriaceae and Ruminococcaceae. The latter family may include *Faecalibacterium prausnitzii* and *Ruminococcus* spp. *F. prausnitzii* is one of the most common inhabitants of the human gut, and it is associated with protection against CD in both humans (71), and in the 2,4,6-trinitrobenzene sulphonic acid (TNBS) induced mouse model (71), although it is often not found in the gut from laboratory mice from commercial breeders (72). Several other *Ruminococcus* spp. have been associated with a low abundance in people with IBD (73). In the horse gut, the abundance of unclassified Ruminococcaceae correlates to a low expression of the T helper cell type 17 produced pro-inflammatory cytokine IL-17 (67), which is a key element in CD (74). Peptostreptococcaceae appear to be over-represented in the guts of colorectal cancer patients (75). Heliobacteriaceae require organic carbon sources; some can convert light energy into chemical energy (76), although the role in IBD seems unclear. *Alistipes* spp. belonging to the phylum Bacteroidetes were significantly decreased in abundance in the KO mice. *Alistipes* spp. are known to strongly impact metabolite formation, such as butyrate and propionate, in the gut (75). A significant increase in the abundance of *Lactobacillus* spp. in meat-fed mice was recorded (Figure 7D). This broad group of bacteria is known for their beneficial effects connected to production of lactic acid and short chain fatty acids (77). Some of them, e.g., *L. rhamnosus*, are anti-inflammatory (78). Others produce specific aminopeptidases, such as *L. brevis*, which produce arginine specific aminopeptidase (79). As we do not have an identification to a species level, we have no explanation, why the *Lactobacillus* abundance is high in meat fed mice, but it is reasonable to assume that it is mainly caused by species capable of decomposition of meat components as both meat and casein are animal sources of the protein in the diets of WT and KO mice.

## Conclusion

To identify potential diet-gut microbiome-host genetics and/or immune systems interactions relevant for patients with IBD we investigated the murine Abcb1a deficient colitis model supplementing the diet with casein or red meat. Our omics-based analysis of intestinal tissue and urine, and associated gut microbiota changes, indicate that not only proteomes in the colon, but also in ileum and urine, are useful for characterizing animal colitis model. In addition, neutrophil degranulation-associated NETs protein components were found elevated from the colon to urine, supporting the potential of NETs as a biomarker for disease assessment. The microbiome of the KO colitis model differs remarkably from the WT microbiome indicating a high impact on the efflux transporter function and occurrence of IBD-related strains.

## Data availability statement

The datasets presented in this study can be found in online repositories. The names of the repository/repositories and accession number(s) can be found in the article/Supplementary material.

## Ethics statement

The study was approved by the Danish Animal Experiments Inspectorate (license number 2012-15-2,934-00089) and was supervised by the in-house Animal Welfare Body of the National Food Institute at the Technical University of Denmark.

## Author contributions

UV, GR-H, and VA designed the study. AM and GR-H collected the material and performed histological assessments. AM performed histological examination of colon biopsies. AS and VA wrote the first draft of the manuscript. AS performed proteomic and immune assay data acquisition. MR, CB, AS, and AF performed microbiota analysis. AS, CA, AH, and TB analyzed the data. AS, TB, LK, AH, UV, AM, and GR-H participated in the manuscript writing. All authors contributed to the article and approved the submitted version.

## Funding

The Erichsen Family Memorial Foundation (Familien Erichsens Mindefond) is acknowledged for grants enabling the animal study (VA). The Danish National Mass Spectrometry Platform for Functional Proteomics (PRO-MS; grant no. 5072-00007B), The Obel Family Foundation; the SparNord Foundation and the Svend Andersen Foundation are acknowledged for grants to the analytical platform enabling parts of this study.

## Conflict of interest

VA is receiving compensation as a consultant for MSD (Merck) and Janssen and advisory board member for MSD (Merck). AH declares industrial collaboration and support as described on https://ivh.ku.dk/english/employees/?pure=en/persons/107126.

The remaining authors declare that the research was conducted in the absence of any commercial or financial relationships that could be construed as a potential conflict of interest.

## Publisher’s note

All claims expressed in this article are solely those of the authors and do not necessarily represent those of their affiliated organizations, or those of the publisher, the editors and the reviewers. Any product that may be evaluated in this article, or claim that may be made by its manufacturer, is not guaranteed or endorsed by the publisher.

## Footnotes

^1^ www.frida.fooddata.dk

^2^ http://proteomecentral.proteomexchange.org

## Abbreviations

ABC: ATP-binding cassette
IBD: Inflammatory Bowel Disease
NETs: Neutrophil extracellular traps
UC: ulcerative colitis
CD: Crohn’s disease
sPCA: sparse principal component analysis
sPLS-DA: sparse Partial Least Squares-Discriminant Analysis model
KO: knockout
WT: wild type
